# The impact of *FADS *genetic variants on ω6 polyunsaturated fatty acid metabolism in African Americans

**DOI:** 10.1186/1471-2156-12-50

**Published:** 2011-05-20

**Authors:** Rasika A Mathias, Susan Sergeant, Ingo Ruczinski, Dara G Torgerson, Christina E Hugenschmidt, Meghan Kubala, Dhananjay Vaidya, Bhoom Suktitipat, Julie T Ziegler, Priscilla Ivester, Douglas Case, Lisa R Yanek, Barry I Freedman, Megan E Rudock, Kathleen C Barnes, Carl D Langefeld, Lewis C Becker, Donald W Bowden, Diane M Becker, Floyd H Chilton

**Affiliations:** 1 Division of General Internal Medicine, Department of Medicine, The GeneSTAR Research Program, The Johns Hopkins University, 1830 E. Monument St., Baltimore, MD 21224, USA; 2 Division of Allergy and Clinical Immunology, Department of Medicine, The Johns Hopkins University, 1830 E. Monument St., Baltimore, MD 21224, USA; 3 Department of Biochemistry, Medical Center Blvd., Wake Forest University Health Sciences, Winston-Salem, NC 27157, USA; 4 Wake Forest Center for Botanical Lipids and Inflammatory Disease Prevention, Medical Center Blvd., Wake Forest University Health Sciences, Winston-Salem, NC 27157, USA; 5 Department of Biostatistics, Bloomberg School of Public Health, Johns Hopkins University, 615 N. Wolfe Street, Baltimore, MD 21224, USA; 6 Department of Human Genetics, 920 E. 58th St., University of Chicago, Chicago, IL 60637, USA; 7 Center for Diabetes Research, Wake Forest University Health Sciences, Medical Center Blvd., Winston-Salem, NC 27157, USA; 8 Division of Public Health Sciences, Department of Biostatistical Sciences, Medical Center Blvd., Wake Forest University Health Sciences, Winston-Salem, NC 27157, USA; 9 Department of Physiology/Pharmacology, Wake Forest University Health Sciences, Medical Center Blvd., Winston-Salem, NC 27157, USA; 10 Department of Internal Medicine, Wake Forest University Health Sciences, Medical Center Blvd., Winston-Salem, NC 27157, USA; 11 Center for Human Genomics, Wake Forest University Health Sciences, Medical Center Blvd., Winston-Salem, NC 27157, USA

## Abstract

**Background:**

Arachidonic acid (AA) is a long-chain omega-6 polyunsaturated fatty acid (PUFA) synthesized from the precursor dihomo-gamma-linolenic acid (DGLA) that plays a vital role in immunity and inflammation. Variants in the Fatty Acid Desaturase (*FADS*) family of genes on chromosome 11q have been shown to play a role in PUFA metabolism in populations of European and Asian ancestry; no work has been done in populations of African ancestry to date.

**Results:**

In this study, we report that African Americans have significantly higher circulating levels of plasma AA (p = 1.35 × 10^-48^) and lower DGLA levels (p = 9.80 × 10^-11^) than European Americans. Tests for association in N = 329 individuals across 80 nucleotide polymorphisms (SNPs) in the Fatty Acid Desaturase (*FADS*) locus revealed significant association with AA, DGLA and the AA/DGLA ratio, a measure of enzymatic efficiency, in both racial groups (peak signal p = 2.85 × 10^-16 ^in African Americans, 2.68 × 10^-23 ^in European Americans). Ancestry-related differences were observed at an upstream marker previously associated with AA levels (rs174537), wherein, 79-82% of African Americans carry two copies of the G allele compared to only 42-45% of European Americans. Importantly, the allelic effect of the G allele, which is associated with *enhanced *conversion of DGLA to AA, on enzymatic efficiency was similar in both groups.

**Conclusions:**

We conclude that the impact of *FADS *genetic variants on PUFA metabolism, specifically AA levels, is likely more pronounced in African Americans due to the larger proportion of individuals carrying the genotype associated with increased FADS1 enzymatic conversion of DGLA to AA.

## Background

Levels of unsaturated fatty acids influence key cellular functions necessary for life including the fluidity of membranes, the function of membrane-associated proteins, signal transduction events, the transcription of proteins and the synthesis of lipid-derived bioactive molecules. Seminal studies published in the 1930's by Burr, Burr and Miller [1] demonstrated that the medium chain unsaturated fatty acid (MC-PUFA), linoleic acid (C18:2,ω6; LA), is essential for the survival and health of rats. Later studies demonstrated that LA is 'essential' because it cannot be synthesized *de novo *in higher animals [2,3], and it is therefore indispensible in the synthesis of the long chain polyunsaturated fatty acids (LC-PUFA), such as arachidonic acid (AA) [4].

The conversion of LA to AA occurs through the actions of two desaturase (Δ6 and Δ5) enzymes and an elongase enzyme that introduce carbon-carbon double bonds to and increase the chain length (2 carbons) of LA, respectively. The Δ5 and Δ6 desaturase enzymatic steps have been recognized to be rate-limiting in AA biosynthesis from LA [5]. In carnivores including humans, AA can also be obtained preformed in animal products, specifically in organ and muscle meats and egg yolks. Once formed or ingested from the diet, AA impacts normal and patho-physiologic immune responses through a variety of mechanisms including its capacity to be converted to potent bioactive products (such as prostaglandins, thromboxanes, leukotrienes and lipoxins), to regulate and activate cellular receptors and to impact the expression of genes that control immune responses [6-8]. In humans, AA constitutes 5-10% of the total fatty acids within inflammatory cellular lipids [9].

The Western diet has undergone a marked change over the past 75 years [10]; in particular, the composition of fats in our diets has changed dramatically. The consumption of LA has increased to an average of 15-20 g/day primarily derived from vegetable oils and margarines. In fact, > 90% of total PUFAs in a typical Western diet is LA. Until recently, biochemical studies using stable isotope studies in subjects largely of European ancestry have indicated only a small proportion of dietary LA is converted to AA in humans suggesting that even in the presence of high LA, there is limited capacity for it to be converted to AA [11-13]. The low rate of conversion was assumed to apply to all human populations equally [5]. However, studies over the past five years suggest genetic variability in the rate of conversion of LA to AA [14-18]. Importantly, certain genetic variants appear to be associated with higher levels of AA, systemic inflammation and inflammatory disorders.

Marquardt *et al *reported the presence of three FA desaturase (*FADS*) gene family members on chromosome 11q12-13 in humans that appeared to be necessary for the synthesis of LC-PUFAs [19]. *FADS1 *and *FADS2 *were demonstrated to encode for Δ5 desaturase and Δ6 desaturase, respectively, and have been demonstrated to be central in the conversion of LA to AA. *FADS1-3, *potentially arising evolutionarily by gene duplication, have a high degree of sequence identity (62-70%), almost identical intron/exon organization [19] and appear to be highly conserved between species (Additional File 1). Numerous studies have examined the effects of genetic variants in *FADS1 *and *FADS2 *in PUFA metabolism in populations of European or Asian descent ({Malerba, 2008 #998;Xie, 2008 #999;Schaeffer, 2006 #1000;Rzehak, 2009 #1002;Bokor, #1004;de Antueno, 2001 #1005} and reviewed in [20]). The strongest genome-wide association (GWAS) signal associated with PUFA levels has been the single nucleotide polymorphism (SNP) rs174537 (p = 5.95 × 10^-46^, [21]). This SNP accounts for up to 19% of the variation in AA levels and maps to an open reading frame (*C11orf9*) 14.4 kb upstream of *FADS1*.

To date, few studies have examined the impact of ancestry on LC-PUFA synthesis and levels. In 1991, Horrobin and colleagues [22] measured AA levels in plasma phospholipids of nineteen subjects from Zimbabwe Africa and found them to be approximately 2-fold higher than a much larger group (N = 458) of subjects with European ancestry. In the current study, we measured levels of circulating ω6 PUFAs in African Americans and European Americans in order to examine ancestry-related differences in PUFA metabolism with respect to the *FADS *loci.

## Results

### Plasma fatty acid profiles differ in Americans of African and European descent

**Figure 1:**
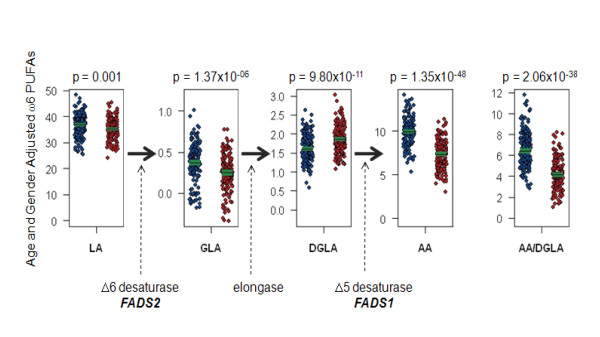
**Population-based Differences in Plasma Fatty Acid Concentrations**. Trait distribution differences between African American (N = 174) and European American (N = 155) individuals from the GeneSTAR study showing distributions in age- and gender-adjusted ω6 PUFAs (LA, GLA, DGLA and AA) and FADS1 enzymatic efficiency (AA/DGLA). Each sample is represented by a single dot in blue for African Americans and red for European Americans. Sample means and confidence interval for the sample mean are presented as the horizontal black line and surrounding green box, respectively. Genes known to play a pivotal role in the desaturation and elongation step in the metabolism of AA are illustrated. Individual PUFAs were expressed as percent of total fatty acids in a sample, and the ratio of AA mass/DGLA mass was calculated as a measure of FADS1 enzymatic efficiency.

Figure 1 shows the distribution of ω-6 PUFAs in the African American and European American adults from the GeneSTAR study. With the exception of DGLA, ω-6 PUFAs examined all appeared to be significantly higher in the African Americans compared to European Americans. There was an increase in the magnitude of the difference between the two racial groups as the length of the carbon chain of the PUFAs increased (p-value for LA = 0.001, for GLA = 1.37 × 10^-06^, for DGLA = 9.80 × 10^-11 ^and for AA = 1.35 × 10^-48^). Furthermore, the ratio of FADS1 product to precursor (AA/DGLA) was markedly higher in the African American subjects (p = 2.06 × 10^-38^) suggesting a differential ability to convert DGLA to AA through FADS1 pathway between the two groups.

### *FADS *SNP frequencies differ in Americans of African and European descent

We compared allele frequencies between the African Americans and European Americans in GeneSTAR for seven of the most highly replicated *FADS *SNPs [16,21] in the region of association with LC-PUFAs. The allele associated with *increased *levels of LC-PUFAs was typically higher in frequency in the African Americans (Table 1). For example at rs174537, the SNP with the strongest published evidence for association with LC-PUFAs, the frequency of the G allele was 91% in the African Americans and only 67% in the European Americans. These differences in allele frequency noted in the GeneSTAR subjects were similarly observed in the DHS study subjects and further replicated in the publicly available data from the International HapMap Project (http://www.hapmap.org) (Table 1).

**Table 1:** **Allelic Frequencies *FADS *Gene Cluster Variants Differ by Race**.

SNP		Allele associated with increased LC-PUFAS	**GeneSTAR**^***a***^		**DHS**^***b***^		**HapMap**^***c***^		
**[ancestral/derived allele]**^***d***^	Position		African American	European American	African American	European American	CEU	ASW	YRI
rs174537 [T/G]	61309256	G	0.91	0.67	0.89	0.65	0.66	0.92	0.99
rs102275 [A/G]	61314379	A	0.37	0.67	0.33	0.64	0.65	0.39	0.31
rs174546 [T/C]	61326406	C	0.92	0.67	0.91	0.65	0.66	0.92	0.99
rs174556 [C/T]	61337211	C	0.92	0.71	0.91	0.68	0.7	0.93	0.99
rs1535 [G/A]	61354548	A	0.86	0.67	0.83	0.64	0.66	0.88	0.88
rs174576 [A/C]	61360086	C	0.74	0.66	0.7	0.64	0.66	0.71	0.72
rs174579 [C/T]	61362189	C	0.95	0.79	0.92	0.79	0.77	0.95	0.99

Frequencies of the allelic variant associated with higher levels of LC-PUFAs at seven SNPs mapping to the *FADS *gene cluster in the family-based GeneSTAR study, subjects from the DHS study, and the publicly availably HapMap project, illustrating higher frequencies in individuals of African ancestry.

^*a*^Allele frequency estimates were obtained from in a defined set of all founders from the GeneSTAR families (339 African American founders and 484 European American founders).

^*b*^Frequency based on 33 independent African American and 89 independent European American subjects from the DHS.

^*c*^Data on the Utah residents with Northern and Western European ancestry from the CEPH collection (CEU), Yoruban in Ibadan, Nigeria (YRI) and African ancestry in Southwestern USA (ASW) from the publicly available International HapMap Project (www.hapmap.org).

^*d*^Definition of ancestral/derived allele based on dbSNP definitions (http://www.ncbi.nlm.nih.gov/projects/SNP/).

**Figure 2:**
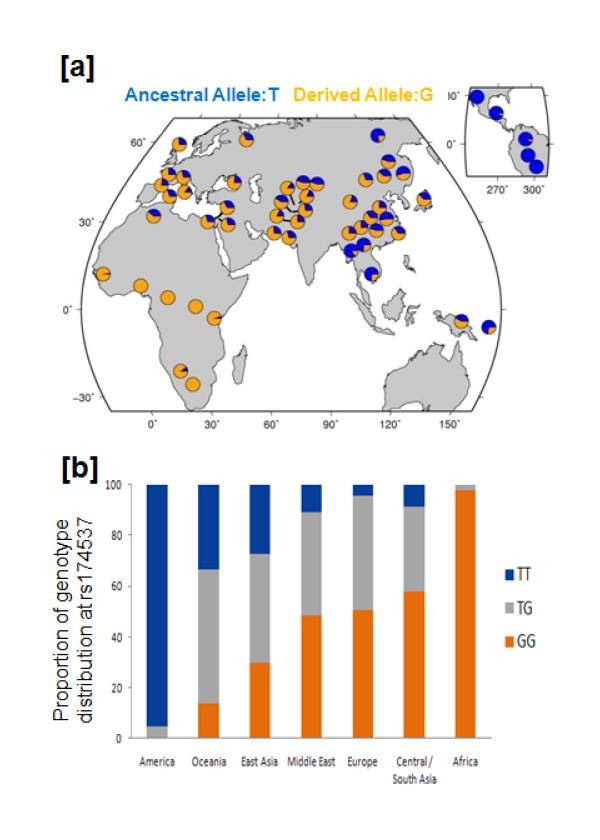
**Geographic Differences in the Allelic Frequencies of a *FADS *Gene Cluster SNP**. Geographic distribution (**a**) of derived allele frequencies (shown in orange) for rs174537 in the 52 populations represented in the Human Genome Diversity Panel Data. The data suggest the fixation of derived rs174537 G allele within the African continent. Similarly, the continental distribution for rs174537 genotypes (**b**) shows a dramatic shift in the predominant homozygous state from Africa to the Americas.

To evaluate variation in the global distribution across these loci, allele frequencies for SNPs in this chromosome region were subsequently examined in the publicly available Human Genome Diversity Panel Data (http://hgdp.uchicago.edu/cgi-bin/gbrowse/HGDP/). We found the increased allele frequencies noted in the admixed African ancestry populations (i.e. African Americans from DHS and GeneSTAR, Table 1) to be more pronounced in the non-admixed populations within Africa. Most noteworthy is the variation at rs174537, where the derived allele (G) has swept to fixation (i.e. 100%) within the African continent, but is at intermediate frequencies in the European and Asian continents and very minimally observed in Central America (Figure 2a). This translates to a 97.5% prevalence of the GG genotype, the genotype associated with *enhanced *AA levels in the African populations in the HGDP, as compared to 50.6% in the European and 0% in the Central American populations in the HGDP (Figure 2b).

### Genotypic Effect of rs174537 on ω6 PUFAs and FADS1 enzymatic efficiency

**Figure 3:**
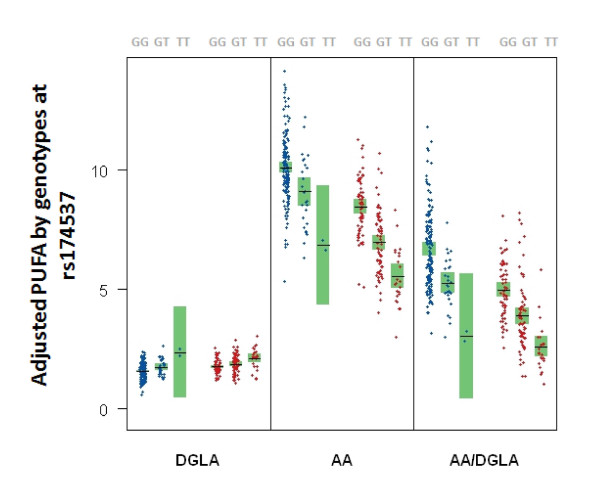
**Plasma Fatty Acid Concentrations Vary by Genotype at rs174537**. Trait distribution differences between African Americans (N = 174) and European Americans (N = 155) participating in the GeneSTAR study, showing genotypic differences in according to DGLA, AA, and AA/DGLA phenotype at marker rs174537 by race. Each sample is represented by a single dot in blue for African Americans and red for European Americans. Sample means and confidence interval for the sample mean are presented as the horizontal black line and surrounding green box, respectively.

Figure 3 and Table 2 summarize the distributions of DGLA, AA, and AA/DGLA by genotype at rs174537 consistent with other published studies [16,21], and show strong genotypic differences in trait means for both racial groups (p = 2.23 × 10^-04 ^- 29.28 × 10^-23^). As shown in Figure 3, the common allele (G) is associated with an *increase *in the mean plasma AA level that is consistent with an additive model in both racial groups. In contrast to AA, for DGLA the common allele (G) was associated with a *decrease *in mean levels that is also consistent with an additive model in both racial groups. For the ratio of AA/DGLA we observed that the common allele (G) appears to be associated with *increased *trait mean, *i.e. *increased enzymatic efficiency. For each of the three traits, the estimate allelic effect (i.e. β in Table 2) has highly overlapping confidence intervals between the two racial groups indicating that the allelic effect on trait distribution is not different between the two ancestry groups. Similar results were noted across all significant SNPs from Table 1 (see Additional File 2).

**Table 2:** **Allelic Effect on Trait Distribution in the Two Ancestry Groups**.

PUFA	Group	p-value	allelic effect β (95% CI)
**DGLA**	African American	2.23 × 10^-04^	0.225 (0.105,0.344)
	European American	9.53 × 10^-05^	0.162 (0.081,0.244)

**AA**	African American	3.72 × 10^-05^	-1.133 (-1.671,-0.594)
	European American	9.28 × 10^-23^	-1.461 (-1.754,-1.170)

**AA/DGLA**	African American	6.07 × 10^-15^	-1.483 (-1.855,-1.110)
	European American	6.34 × 10^-19^	-1.172 (-1.431,-0.914)

Allelic effect on trait distributions of age- and gender-adjusted ω6 PUFAs (AA and DGLA) and FADS1 enzymatic efficiency (AA/DGLA) was examined in the African Americans and European Americans from the GeneSTAR Study at marker rs174537.

### Tests for association between variants on chromosome 11q13 and ω6 PUFAs and FADS1 enzymatic activity

**Figure 4:**
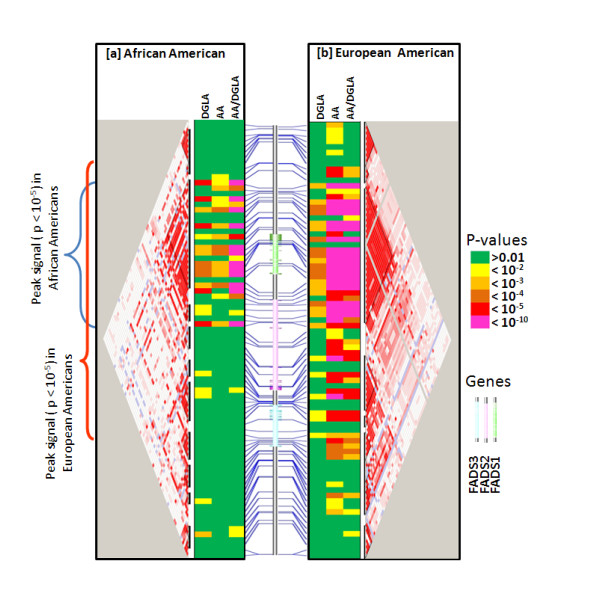
**Peak Association by Race**. Tests for association for AA, DGLA and FADS1 enzymatic activity (AA/DGLA) in the ascertained sample from GeneSTAR in African Americans and European Americans for 100 SNPs on chromosome 11q13. Strength of Association depicted as in the legend, LD patterns (low to strong ranging from white to red) along with LD blocks (black triangles), and physical location of SNPs (blue lines) relative to the three *FADS *genes (see insert).

With the availability of genome-wide genotype data, tests for association were performed across 80 SNPs in a 184 kb region on 11q13 in the GeneSTAR subjects stratifying on race. Association tests were performed for DGLA, AA and AA/DGLA, the three traits found to be most different between the two racial groups and that appear to highlight FADS1 enzymatic activity as one reason for this difference. Focusing on p-values < 10^-5 ^in Figure 4, the association signal in the African American subjects spanned a genomic region of 58 kb (blue bracket) which was half of that observed in the European Americans (~121 kb, red bracket) and reflective of smaller LD blocks and less inter-SNP correlation in the African Americans. In the African Americans, we observed a total of thirteen LD blocks, which included 60 out of the 80 total SNPs and these encompassed a total 85.51 kb of the 184 kb region. In the European Americans, we observed fewer (N = 10) LD blocks, but these included more (N = 69) SNPs and encompassed a larger region (121 kb).

The peak association signal in the African American sample from GeneSTAR for DGLA was p = 1.34 × 10^-10 ^(rs968567, explains 12% trait variation), for AA was p = 3.72 × 10^-5 ^(rs174537, explains 10% of trait variation), and for AA/DGLA was p = 2.85 × 10^-16 ^(rs174534, explains 15% trait variation). In the European American group, maximum association signal noted was p = 7.84 × 10^-5 ^(rs174549, rs174555, rs174556, explains 11% trait variation) for DGLA, p = 2.68 × 10^-23 ^(also at rs174549, rs174555, rs174556, explains 39% trait variation) for AA, and 3.63 × 10^-19 ^(rs174568, explains 29% trait variation) for AA/DGLA. With the exception of the signal at DGLA (rs968567) in the African Americans, the strongest signal in both groups was in the largest LD block of each group, which includes all of the *FADS1 *gene.

## Discussion

This is the first report comparing plasma levels of AA and DGLA and genotypes at the FADS locus in African Americans and European Americans. Our data suggest that there are similar rates of conversion of the precursor DGLA to its product AA among African Americans and European Americans for each genotype at the lead SNP rs174537 in the FADS locus. However, strong allele frequency differences between the two groups, results in a higher proportion of African Americans carrying the genotypes known to be associated with higher levels of AA and AA/DGLA, i.e. the GG genotype associated with more efficient conversion of DGLA to AA.

Based on allele frequency estimates from the GeneSTAR and DHS studies, 79-82% of African Americans carry two copies of the G allele while only 42-45% of European Americans are GG. While it is possible that African Americans consume higher quantities of preformed AA itself, the most striking racial differences in ω-6 PUFAs levels in these GeneSTAR data are with DGLA, AA and AA/DGLA, a measure of FADS1 enzymatic activity. This coupled with the establishment of identical FADS1 enzymatic efficiency (measured by AA to DGLA ratios) with respect to key *FADS *genetic variants plus the difference in proportion of population carrying the GG genotype, strongly suggest that part of the striking phenotypic differences between the two racial groups are genetic in origin.

The GeneSTAR African American group appears to confirm the association signal detected in European Americans from previously published reports. However, patterns of linkage disequilibrium are considerably different between the two racial groups, and what appears to be a region extending over ~121 kb with association p-values as strong as 10^-5 ^in the European Americans appears less than half (~58 kb) that size in the African Americans. The regions with peak signal in both groups include rs174537, the peak GWAS SNP identified by Tanaka *et al *[21], as well as rs968567, the SNP identified by Lattka *et al *[23] as a locus modulating the complex regulation of *FADS2 *gene transcription. However, the signal at rs968567 does not appear to be the peak association signal in either racial group, with the exception of the DGLA phenotype in African Americans. Our work focusing on FADS1 enzymatic efficiency suggests that there may be other loci accounting for the association signals in this region.

## Conclusions

Given our observations of: (i) increased levels of AA in African American subjects; (ii) a similar effect of genotype on FADS1 enzymatic efficiency in both racial groups; and (iii) increased frequencies of alleles associated with higher levels of AA in African Americans in the *FADS *cluster, we surmise that African Americans are genetically predisposed for more efficient conversion of MC-PUFAs to LC-PUFAs. The pattern of allele frequency in the *FADS *gene cluster between the African American and European American subjects from both the DHS and GeneSTAR data were also observed in the HapMap samples, and were magnified when examined across the full set of HGDP subjects. We noted a pattern of allele frequency differences unique to the populations within the African continent across multiple SNPs in this region (data not shown) and highlight that, for a peak SNP in this region (rs174737), the allele that is associated with increased AA levels is in fact fixed in African populations, and within continental Africa, 97.5% of individuals carry the GG genotype based on the HGDP.

There remains a disproportionate burden of preventable disease, death, and disability among racial and ethnic minority populations, especially African Americans. Observed racial and ethnic differences in prevalence and/or severity of common diseases can only be explained in part by environmental, social, cultural, or economic factors, and genetic factors are likely at play [24]. Differences in the prevalence and severity of chronic diseases involving inflammation are further corroborated by differences in inflammatory biomarkers [25,26]. If the *FADS *locus does indeed account for as much as 10% of the total phenotypic variation in plasma AA levels in African Americans and as high as 39% in European Americans, as seen in our study, then the implications for a transition to a diet enriched in LA and AA (i.e., a Western diet) and the consequent impact on inflammation through AAs capacity to be converted to potent bioactive products [6-8] might be quite significant. Over 90% of PUFA consumed in the typical Western diet is LA, and the dramatic increase in LA consumption to 15-20 g/day that has occurred over the past five decades in developed countries would be much more likely to impact AA levels in populations of African ancestry than European ancestry given the differences in genotype frequencies. We further conclude from our work that the need to understand the genetics of PUFA metabolism both at the *FADS *locus and elsewhere in the genome is important. Humans of different ancestry appear to exhibit differences in the rate of conversion of LA to AA, and this may result in differences in inflammatory contributions to chronic diseases as discussed above.

## Methods

### Study Populations

#### Genetic Study of Atherosclerosis Risk (GeneSTAR)

As previously described [27], the GeneSTAR study included subjects from European American and African American families identified through a proband with documented coronary artery disease prior to 60 years of age. Apparently healthy siblings of the probands, the adult offspring of both the probands and their siblings, and the coparents of the offspring were recruited between 1998 and 2002. Genotype data were available on all subjects from GeneSTAR, including 1,091 African Americans and 1,625 European Americans from 306 and 467 families, respectively. The final selected sample comprised African Americans (N = 174, 61% male; mean age = 38.0 ± 12.2) from 98 families and European Americans (N = 155, 32% male; mean age = 38.6 ± 12.1) from 126 families. Majority of the families were singletons (N = 149) and 75 had two more individuals (median = 2, range 2 - 8). The study protocol was approved by the Johns Hopkins School of Medicine IRB, and all subjects gave written informed consent.

#### Diabetes Heart Study (DHS)

There were 229 individuals from the Diabetes Heart Study (DHS [28]) available for genotyping that included European American (N = 166; from 89 families) and African American (N = 63; from 33 families) subjects with metabolic syndrome. Methods for ascertainment and recruitment for the DHS have been described previously [28]. The Wake Forest University School of Medicine Institutional Review Board (IRB) approved study protocols, and all participants provided written informed consent.

### Fatty Acid Analysis

Plasma was isolated from fasting whole blood samples and fatty acid methyl esters (FAME) were prepared [29] after saponification from duplicate samples (100 μl) in the presence of an internal standard (triheptadecanoin) as described in detail elsewhere [30]. A panel of 23 fatty acids was quantified by gas chromatography with flame ionization detection. Individual ω6 fatty acids (18:2ω6 linoleic acid = LA, 18:3ω6 gamma linoleic acid = GLA, 20:3ω6 dihomo-gamma linolenic acid = DGLA and 20:4ω6 arachidonic acid = AA) were expressed as percent of total fatty acids in a sample, and the ratio of AA mass/DGLA mass was calculated as a measure of FADS1 enzymatic efficiency.

### Genotyping and Analysis of Allele Frequencies

In GeneSTAR, genome-wide SNP genotyping was performed at deCODE Genetics, Inc. using the Human 1Mv1_C array from Illumina, Inc. where 1,044,977 markers were released with an average call rate per sample of 99.65% and an overall missing data rate of 0.35%. From this panel, a total of 100 SNPs that mapped to a 184 kb region on chromsome 11q13, and encompassing *C11orf9, FADS1, FADS2 *and *FADS3 *were available, and a subset of 80 SNPs with a minor allele frequency (MAF) > 5% in at least one of the two ethnic groups was used in the final analyses. PLINK [31] was used to detect and remove Mendelian errors. Hardy-Weinberg equilibrium (HWE) and MAF for each SNP was tested in a defined set of independent subjects (N = 319 African American and 484 European American) representing the founders of the pedigrees. The highest deviation from HWE was at a p-value of 0.0154, well above a threshold of of 0.0006 (i.e. p = 0.05/number of SNPs tested in each group).

In the DHS population, seven SNPs mapping to the *FADS *gene cluster (rs174537, rs102275, rs174546, rs174556, rs1535, rs174576, rs174579) were selected based on previous publications [16,21]. Genotypes were determined using a Sequenom Mass ARRAY SNP genotyping system (Sequenom Inc., San Diego, CA, USA) [32]. Of the samples, 3.5% were genotyped in duplicate with 100% reproducibility across the SNPs.

### Statistical Analyses and tests for association

In the GeneSTAR population, the levels of five ω6 fatty acids (LA, GLA, DGLA, AA and AA/DGLA) were adjusted for age and gender and analyzed to test for differences between race and for an association with each individual SNP within each racial group. Association was performed under an additive model (i.e. a linear recoding of the SNP as 0/1/2 for 0, 1 and 2 copies of the minor allele) for AA, DGLA and AA/DGLA. Regression models were implemented in the generalized estimating equation (GEE) framework with an exchangeable covariance matrix to correct for familial correlation [33]. Analyses were carried out using R (v.2.11.1) with the gee package. Principal components-based estimates of admixture were obtained using the smartpca program in EIGENSOFT [34]. The first two eigenvectors in the European Americans and the first eigenvector in African Americans were included in the regression models. Linkage disequilibrium (LD) was assessed by calculating D' and r^2 ^within Haploview [35], relying on a set of independent individuals in the data. Haplotype blocks were defined according to the algorithm of Gabriel *et al *[36]. Tests for association were evaluated at a stringent Bonferroni threshold of 0.0002 within each racial group (*i.e.*, a = 0.05/(80 SNPs * 3 traits)).

## Abbreviations

AA: arachidonic acid; DGLA: dihomo-gamma-linolenic acid; FADS: fatty acid desaturase; FAME: fatty acid methyl ester; GEE: generalized estimating equation; GLA: gamma-linolenic acid; GWAS: genome-wide association study; HGDP: Human Genome Diversity Panel; HWE: Hardy-Weinberg equilibrium; LA: linoleic acid; LC: long chain; LD: linkage disequilibrium; MAF: minor allele frequency; MC: medium chain; PUFA: polyunsaturated fatty acid; SNP: single nucleotide polymorphism.

## Authors' contributions

RAM, FCH and KCB generated the hypotheses, designed the experiments; RAM LRY and MK performed analysis allele frequencies. RAM and DGT performed analysis on the HGDP data; CEH performed SNP genotyping and association analyses; MER selected SNPs and designed the genotyping assays; SS and PI performed fatty acid analyses; IR, DV, BS, LDC, JTZ and CDL provided statistical and genetic analyses. DWB and BIF provided access to DHS study data; LCB and DMB provided access to GeneSTAR data; RAM and FCH prepared the manuscript; SS, KCB, DGT, CEH, DWB assisted in manuscript preparation. All authors have read and approved the final manuscript.

## Supplementary Material

Additional File 1 **Conservation of *FADS *gene cluster presented as percentage homology for protein and DNA between humans and other species as reported in Homologene (http://www.ncbi.nlm.nih.gov/homologene) revealing considerable conservation in range of species including chicken for *FADS1*(~737%) and *FADS2 *(~75%), and zebra fish for *FADS2 *(~65%)**. Click here for file

Additional File 2 Allelic Effect on Trait Distribution in the Two Ancestry Groups. Allelic effect on trait distributions of age- and gender-adjusted ω6 PUFAs (AA and DGLA) and FADS1 enzymatic efficiency (AA/DGLA) was examined in the African Americans and European Americans from the GeneSTAR Study Click here for file
