# Supplementary material for: The impact of FADS genetic variants on ω6 polyunsaturated fatty acid metabolism in African Americans
Source: BMC Genet. 2011 May 20;12:50. doi: 10.1186/1471-2156-12-50 (PMC3118962; doi:10.1186/1471-2156-12-50)
Supplement: Additional File 1 — Conservation of FADS gene cluster presented as percentage homology for protein and DNA between humans and other species as reported in Homologene (http://www.ncbi.nlm.nih.gov/homologene) revealing considerable conservation in range of species including chicken for FADS1(~737%) and FADS2 (~75%), and zebra fish for FADS2 (~65%). [file 1471-2156-12-50-S1.DOC]

**Additional File 1**

Conservation of *FADS* gene cluster presented as percentage homology for protein and DNA between humans and other species as reported in Homologene (<http://www.ncbi.nlm.nih.gov/homologene>) revealing considerable conservation in range of species including chicken for *FADS1*(~737%) and *FADS2* (~75%), and zebra fish for *FADS2* (~65%).

| **Homo sapien vs.** | **human vs.** | ***FADS1*** | | ***FADS2*** | | ***FADS3*** | |
| --- | --- | --- | --- | --- | --- | --- | --- |
|  |  | **Protein** | **DNA** | **Protein** | **DNA** | **Protein** | **DNA** |
| **Pan troglodytes** | chimpanzee | 99.8 | 99.8 | 99.8 | 99.8 | 99.8 | 99.7 |
| **Canis lupus familiaris** | dog | 90.8 | 91.5 | 89.8 | 90.7 | 86.4 | 87.2 |
| **Bos taurus** | cattle | 88.2 | 86.6 | 89.6 | 89.6 | 88.5 | 89.1 |
| **Mus musculus** | mouse | 89 | 85.9 | 87.6 | 87.2 | 90.1 | 87.9 |
| **Rattus norvegicus** | rat | 88.5 | 86 | 88.3 | 87.3 | 89.9 | 87.8 |
| **Gallus gallus** | red jungle fowl | 73.2 | 72.4 | 77 | 74.9 | - | - |
| **Danio rerio** | zebrafish | - | - | 64.9 | 67.2 | - | - |
