# Supplementary material for: The impact of FADS genetic variants on ω6 polyunsaturated fatty acid metabolism in African Americans
Source: BMC Genet. 2011 May 20;12:50. doi: 10.1186/1471-2156-12-50 (PMC3118962; doi:10.1186/1471-2156-12-50)
Supplement: Additional File 2 — Allelic Effect on Trait Distribution in the Two Ancestry Groups. Allelic effect on trait distributions of age- and gender-adjusted ω6 PUFAs (AA and DGLA) and FADS1 enzymatic efficiency (AA/DGLA) was examined in the African Americans and European Americans from the GeneSTAR Study [file 1471-2156-12-50-S2.DOC]

**Additional File 2**

**Allelic Effect on Trait Distribution in the Two Ancestry Groups.** Allelic effect on trait distributions of age- and gender-adjusted 6 PUFAs (AA and DGLA) and FADS1 enzymatic efficiency (AA/DGLA) was examined in the African Americans and European Americans from the GeneSTAR Study

|  | **African American** | | | | | |
| --- | --- | --- | --- | --- | --- | --- |
|  | **DGLA** | | **AA** | | **AA/DGLA** | |
| **SNP** | **allelic effect β (95% CI)** | **p-value** | **allelic effect β (95% CI)** | **p-value** | **allelic effect β (95% CI)** | **p-value** |
| **rs102275** | 0.052 (-0.01,0.122) | 0.138 | 0.029 (-0.31,0.376) | 0.869 | -0.19 (-0.56,0.173) | 0.297 |
| **rs1535** | 0.031 (-0.06,0.130) | 0.536 | -0.53 (-1.11,0.036) | 0.067 | -0.43 (-0.87,0.006) | 0.053 |
| **rs174546** | 0.229 (0.112,0.347) | 1.30E-04 | -1.12 (-1.67,-0.58) | 4.27E-05 | -1.50 (-1.86,-1.13) | 1.18E-15 |
| **rs174556** | 0.254 (0.130,0.379) | 5.64E-05 | -1.09 (-1.68,-0.49) | 0.000 | -1.52 (-1.90,-1.14) | 6.53E-15 |
| **rs174576** | 0.129 (0.037,0.222) | 0.006 | -0.22 (-0.66,0.221) | 0.328 | -0.68 (-1.16,-0.20) | 0.005 |
| **rs174579** | 0.276 (0.172,0.379) | 1.68E-07 | -0.97 (-1.52,-0.41) | 0.001 | -1.53 (-1.90,-1.15) | 1.36E-15 |
|  | **European American** | | | | | |
|  | **DGLA** | | **AA** | | **AA/DGLA** | |
|  | **allelic effect β (95% CI)** | **p-value** | **allelic effect β (95% CI)** | **p-value** | **allelic effect β (95% CI)** | **p-value** |
| **rs102275** | 0.149 (0.065,0.233) | 0.001 | -1.42 (-1.71,-1.13) | 5.59E-22 | -1.11 (-1.39,-0.82) | 2.39E-14 |
| **rs1535** | 0.157 (0.079,0.236) | 8.17E-05 | -1.39 (-1.68,-1.10) | 4.81E-21 | -1.13 (-1.38,-0.88) | 8.91E-19 |
| **rs174546** | 0.162 (0.080,0.243) | 9.53E-05 | -1.46 (-1.75,-1.17) | 9.28E-23 | -1.17 (-1.43,-0.91) | 6.34E-19 |
| **rs174556** | 0.176 (0.088,0.263) | 7.84E-05 | -1.47 (-1.76,-1.18) | 2.68E-23 | -1.19 (-1.46,-0.92) | 6.43E-18 |
| **rs174576** | 0.140 (0.060,0.221) | 0.001 | -1.34 (-1.63,-1.06) | 2.64E-20 | -1.05 (-1.32,-0.78) | 2.88E-14 |
| **rs174579** | 0.159 (0.079,0.240) | 1.03E-04 | -0.94 (-1.30,-0.58) | 2.36E-07 | -0.87 (-1.17,-0.57) | 1.57E-08 |

.
